# External Criticism by Parents and Obsessive Beliefs in Adolescents: Mediating Role of Beliefs Associated With Inflated Responsibility

**DOI:** 10.5539/gjhs.v8n5p125

**Published:** 2015-09-16

**Authors:** Zohreh Halvaiepour, Mehdi Nosratabadi

**Affiliations:** 1 Sirjan College of Medical Sciences, Kerman University of Medical Sciences, Kerman, Iran; 2 Department of Health Services Management, Isfahan University of Medical Sciences, Isfahan, Iran

**Keywords:** obsessive-compulsive disorder, Inflated Responsibility beliefs, External Criticism

## Abstract

**Background and Objectives:**

Obsessive-compulsive disorder (OCD) is considered as a rare disorder in children. According to cognitive theories, criticism triggers responsibility behavior and thus causes obsessive behaviors. The purpose of the present study was to investigate the mediating role of beliefs associated with responsibility in the relationship between external criticism of parents and obsessive beliefs in adolescents.

**Materials and Methods:**

In this study, 547 high school students aged from 15 to18 years were selected using multi-stage cluster random sampling from four regions of the education office in Shiraz. Obsessive Beliefs Questionnaire-child version (OBQ-CV), Pathway to Inflated Responsibility beliefs Scale (PIRBS), and perceived criticism questionnaire were used to collect data. Pearson’s correlation was used to investigate the relationship between the study variables. For analysis of mediation model, multiple mediators analysis using Macro Software was used.

**Results:**

External criticism only indirectly and through beliefs associated with inflated responsibility accounts for 6% of the variance of responsibility, 14% of the variance of threat estimation and 10% of the variance of perfectionism of obsessive beliefs (P<0.05). However, external criticism, both directly and indirectly and through beliefs associated with inflated responsibility accounts for 7% of the variance of the importance of obsessive beliefs.

**Conclusion:**

This study showed that the beliefs associated with inflated responsibility can mediate the relationship between external criticism and obsessive beliefs. According to the cognitive model of Salkovskis, criticism by parents, as a violation to and an influence on children, by affecting the subscales of inflated responsibility, can increase the symptoms of obsessive-compulsive disorder. In order to identify potential affecting mechanisms of criticism on obsessive-compulsive disorder, further experimental research is required.

## 1. Introduction

Obsessive-compulsive disorder (OCD) is characterized by recurrent obsessions and stable thoughts, imaginations and impulses which are accompanied by compulsions intending to reduce or prevent the resulting discomfort (Association, 1994). In individuals with obsessive-compulsive disorder, obsessions are diagnosed with turbulent experiences; compulsions of the disorder include washing, cleaning, inspecting, searching for security, repetitive actions, and covert obsessions such as mental formalities generally to prevent the frightening results or events or to drop disturbance caused by obsessions (WHO, 1999). Epidemiologic studies suggest that OCD is the Fourth most common mental disorder after phobias, substance abuse and major depression (Karno, Golding, Sorenson, & Burnam, 1998). Prevalence rate of obsessive-compulsive disorder lifetime is estimated to be from 2 to 5.5% (Torres & Lima, 2005). Fullana et al estimated that between 21 to 25 percent of the general population have confirmed the signs and symptoms of the disorder (Fullan, Mataix-Cold, & Caspi, 2009). The disorder is more common among children and adolescents compared to previous research on the disorder and often tends to be chronic (Heyman, Fombonne, Simmons, & Ford, 2001). In various studies the prevalence of this disorder is estimated from 1 to 4 percent in children (Douglass & Moffitt, 1995; Rapoport, Inoff-Germain, Weissman, & Greenwald, 2000).

Research on OCD in the past decade was associated with the increasing evidence of cognitive models and effectiveness of cognitive-behavioral therapy (CBT) in both adults and children with OCD. Cognitive theories of obsessive-compulsive disorder are derived from the central role of dysfunctional beliefs in common interventions in the growth and maintenance of obsessive-compulsive disorder. However, research has provided evidence for cognitive theories of adults (Frost & Steketee, 2002). There is little evidence about the role of dysfunctional beliefs in the obsession of children. There are some studies surrounding the association between obsessive-compulsive syndrome and some dysfunctional beliefs, such as over-estimation of threats, heightened responsibility, and perfectionism, intolerance of uncertainty, overpaid attention to threat and importance of thoughts (Barrett & Healy, 2003; Rachman, 1993).

Among cognitive mediations thought to be the basis of the disorder, responsibility, or more accurately the perceived sense of responsibility, have been considered in the literature for nearly 15 years (Rachman, 1998, 2002; P. Salkovskis, 1985; P. M. Salkovskis, Shafran, Rachman, & Freeston, 1999). Patients with OC (Obsessive Compulsive) express an inflated sense of responsibility and according to the importance of this in obsessive-compulsive theories; the Obsessive Compulsive Cognitions Working Group viewed the responsibility beliefs as the first of the six areas related to obsessive compulsive beliefs (Steketee & Frost, 2001). Salkovskis (P. Salkovskis, 1985, 1989) points out that the inflated responsibility beliefs are the central point of obsessive compulsive disorder and if the disturbing thoughts are interpreted in such a way that the person finds him or her responsible for the subsequent damage to himself or herself or others, obsessive pattern is formed.

Salkovskis et al imply that the experience of progressive criticism may lead to increased responsibility (P. M. Salkovskis et al., 1999). Childhood experience of regular criticism from parents or family members can cause turbulence for teenager. Teenagers are afraid of criticism, and it is believed that this fear leads to punishment prevention. Thus, as to prevent they focus on avoidance strategies. Active avoidance strategies entail responsibility to prevent what stop criticizing. Such strategies lead to obsessive behaviors such as inspecting, avoiding the potential threats and avoiding the criticism (P. Salkovskis, 1989).

There are limited evidence on obsessive-compulsive features and communication models in non-clinical populations. Most of these studies focused on middle aged and elderly populations (Essau, Conradt, & Petermann, 2000; Fullana et al., 2010; Shams et al., 2011) and only a few studies have noted school-aged children (Canals, Martínez, Cosi, & Voltas, 2012; Voltas, Martínez, Arija, Aparicio, & Canals, 2014). There have been studies in adults and adolescents in non-Western countries such as Iran (Ghassemzadeh, Bolhari, Birashk, & Salavati, 2005) and in non-clinical samples of adolescents in Turkey (Altın & Gençöz, 2007) as well, and the findings of these studies all support the hypothesis of the role of inflated responsibility in Salkovskis theory.

With regard to the discussed issues it seems that criticism triggers responsibility behaviors and responsibility behaviors on the other hand cause obsessive behaviors. Despite the abundance of research on adults’ OCD, research on the mechanisms involved in the maintenance of the disorder in childhood and adolescence is little. Therefore, the aim of this study is to investigate the mediating role of beliefs related to inflated responsibility in the relationship between external criticism of parents and obsessive-compulsive beliefs of Iranian teenagers.

## 2. Methods

### 2.1 Study Design

This study using a correlation design, examined the relationship between the dimensions of responsibility, the dimensions of obsessive-compulsive symptoms and the external criticism of parents using structural equation modeling techniques.

### 2.2 The Population and Sampling

The study population consisted of all 15 to 18-year-old high school students in Shiraz who were studying during the academic year 2011-2012. Of the population, 547 subjects were selected using multi-stage cluster sampling from four different regions of the Education Office in Shiraz. Sampling was performed as follows: first two of the four regions of the education office in Shiraz, and then 6 high schools from these regions, including three girls high school and 3 boys high school, and 4 classes were randomly selected from each school and they voluntarily responded to the survey questionnaires.

### 2.3 Research Tools

**Obsessive Beliefs Questionnaire child-version (OBQ-CV):** The questionnaire was developed by Coles et al. (M. Coles et al., 2010) and evaluates four subscales of responsibility (R), threat estimation (RT), perfectionism/certainty (PC) and importance/control of thoughts (ICT) in the age group of 8-18 years using 44 questions. The questions are ranked using a 5-degree Likert scale (strongly disagree to strongly agree) and higher score indicates more obsessive beliefs. Walters et al. (Wolters et al., 2011) have examined psychometric properties of the original version of the questionnaire in a German population. The results of the validity assessment of the questionnaire showed that the standard validity is acceptable. Also, the reliability was obtained for clinical and non-clinical groups and different time intervals for the total scale and its subscales ranging from 0.62 to 0.90. In Iran, Halvaeipour et al. (Halvaiepour, Khormaei, Khanzadeh, & Nosratabadi, 2013) also examined psychometric properties of this questionnaire using confirmatory factor analysis (CFA) in the age group of adolescents. The results showed that the four-factor model of the questionnaire of obsessive beliefs have a good fit to the data, and only 6 questions for being insignificant factors were excluded. Also, the results of the combined reliability assessment showed that the reliability for the total scale was 0.84 and for its subscales ranged from 0.72 (for importance/control of thoughts subscale) to 0.87 (for threat estimate subscale).

**Pathways to Inflated Responsibility Beliefs Scale (PIRBS):** Coles and Schofield (M. E. Coles & Schofield, 2008) have developed this scale based on the responsibility beliefs raised by Salkovskis et al. (P. M. Salkovskis et al., 1999). This scale using 23 items assesses four beliefs associated with inflated responsibility, including Heightened Responsibility (HR), Over Protection (OP), Rigid Rules (RR) and actions/inactions caused a serious misfortune (AI/C). Coles and Schofield (M. E. Coles & Schofield, 2008) in their study confirmed validity of this scale using exploratory and confirmatory factor analysis and its concurrent validity using obsessive thoughts questionnaire. Also, results showed that total reliability of this scale was 0.86 using internal consistency and reliability of its four subscales ranged from 0.78 to 0.90 using the same method. In addition, the total reliability of this scale using test-retest was 0.71 and reliability of the four subscales using the same method ranged from 0.58 to 0.79. In Iran, Halvaei Pour and Nosratabadi (Halvaiepour & Nosratabadi, 2014) also studied psychometric characteristics of this scale using confirmatory factor analysis in adolescents. The results of this study showed that the four-factor model of the scale has a good fitness to the data and all factors are significant. Also, the results of combined reliability assessment using confirmatory factor analysis showed that the coefficient for the total scale was 0.61 and for its subscales ranged from 0.60 (for RR subscale) to 0.85 (for AI/C subscale).

**Perceived Criticism Inventory (PCM):** This 6-item questionnaire has been developed by Hooley and Teasdale (Hooley & Teasdale, 1989) using a Likert scale of 1 (no critical) to 10 (very critical), measures external criticism. Test-retest reliability of the questionnaire during 20 successive weeks in two different samples was obtained 0.75. Research results on the validity show that the questionnaire has a good discriminative validity and an acceptable predictive validity in terms of medical outcomes in relation to scales including depression, anxiety and personality traits (Chambless & Steketee, 1999; Hooley & Teasdale, 1989). Reliability and validity of the questionnaire has been studied in Iranian population. Halvaei Pour (Halvaiepour, 2012) studied psychometric properties of the scale in an Iranian population using confirmatory factor analysis in a group of adolescents. The results of this study showed that the one-factor model of the scale has a good fitness to data and all factors are significant. Also, the results of the combined reliability assessment showed that the coefficient for the total scale is equal to 0.63.

### 2.4 Data Analysis

Pearson’s correlation was used to examine the relationship between the study variables. For analysis of mediation model, multiple mediators analysis (Preacher & Hayes, 2008) was used using macro software.

## 3. Results

“Preacher and Hayes” in 2008 introduced Macro software to perform multiple mediators analysis. The software in addition to assess the mediation of some variables in relation to relationships between exogenous and endogenous variables and calculating direct, indirect and total effects of which the basics are consistent with Baron and Kenny path analysis (1986), also assesses the significance of indirect effects using bootstrapping method. Before entering into the results of the analysis, the correlation between the variables of mediation model was evaluated using Pearson correlation. Table 1 shows the results.

**Table 1:** Correlation between variables of the model

		Endogenous variables				Exogenous variable
	
		Importance	Perfectionism	Threat estimation	Responsibility	External criticism
	HR	0.09*	0.12^**^	0.14^**^	0.12^**^	0.16^**^
**Mediator variables**	OP	0.13^**^	0.20^**^	0.26^**^	0.14^**^	0.13^**^
	RR	0.23^**^	0.30^**^	0.28^**^	0.23^**^	0.18^**^
	AI/C	0.15^**^	0.10*	0.28^**^	0.11*	0.20^**^
**Exogenous variable**	External criticism	0.13^**^	0.06	0.06	0.07	**1**

*Note*. HR: Heightened Responsibility, OP:Over Protection, RR: Rigid Rules, AI/C: actions/inactions caused

* P<0.05

** P<0.01

The result provided Table 1 show that there is a positive and significant relationship between external criticism as an exogenous variable and all beliefs associated with inflated responsibility as mediator variables. The results also show that there is a positive and significant relationship between all beliefs associated with inflated responsibility as mediator variables and all aspects of obsessive beliefs as endogenous variables. Finally, the results of Table 1 show that there is only a positive and significant relationship between external criticism and importance of obsessive beliefs dimension.

To assess multiple mediation using Macro Software, first the direct effects of the exogenous variables were investigated on mediator and endogenous. In Table 2, the direct effect of external criticism on beliefs related to inflated responsibility (mediator) and obsessive beliefs (endogenous or dependent) are provided.

**Table 2:** Direct effect of external criticism on beliefs related to inflated responsibility and obsessive beliefs

	External criticism		

		Standard coefficient	sig
beliefs related to inflated responsibility	HR	0.15	**0.001**
	OP	0.18	**0.001**
	RR	0.13	**0.003**
	AI/C	0.20	**0.001**
	Responsibility	0.006	**0.88**
Obsessive beliefs	Threat estimation	-0.04	**0.30**
	Perfectionism	-0.05	**0.25**
	Importance	0.12	**0.008**

As Table 2 results show, the direct effects of external criticism on all aspects of beliefs related to inflated responsibility as the mediator variable are significant; however, the external criticism has only a direct and significant effect on importance of obsessive beliefs aspect.

At the next step, to investigate the mediating role of beliefs associated with inflated responsibility, the effects of these variables on the dimensions of obsessive beliefs were studied. Table 3 shows the coefficient and significance of these effects.

**Table 3:** Effect of beliefs associated with inflated responsibility on the dimensions of obsessive beliefs

	Importance		Perfectionism		Threat estimation		Responsibility	
			
	Standard coefficient	sig	Standard coefficient	sig	Standard coefficient	sig	Standard coefficient	sig
HR	0.02	0.52	0.04	0.27	0.04	0.27	0.05	**0.18**
RR	0.19	0.001	0.25	0.001	0.18	0.001	0.18	**0.001**
OP	0.04	0.27	0.12	0.007	0.15	0.001	0.06	**0.14**
AI/C	0.11	0.02	0.007	0.98	0.19	0.001	0.02	**0.51**

**Figure 1:**
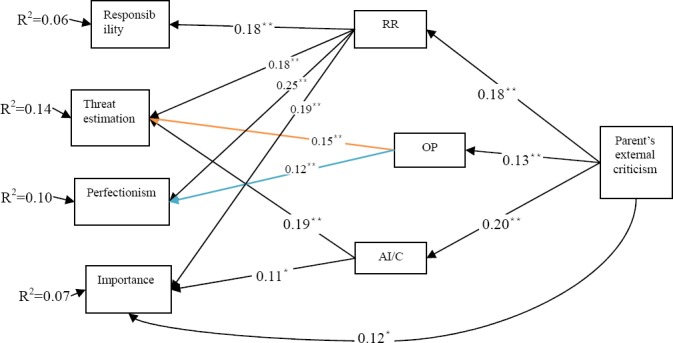
Model of mediation effect of beliefs related to inflamed responsibility in relation to external criticism and obsessive beliefs (P**<0.01, P*<0.05)

Results of Table 3 show that among mediator variables, Rigid Rules have significant and positive effect on all obsessive beliefs, while heightened responsibility has no effect on any significant obsessive beliefs. Figure 1 shows the extracted model of mediation of beliefs related to inflamed responsibility in relation to the relationship between external criticism and obsessive beliefs with regard to the results of Tables 2 and 3 (mediating variables that were not significantly related to dependent or exogenous variables were excluded). Standard coefficient for each variable is shown in the table.

The model presented in Figure 1 shows that external criticism only indirectly and through beliefs associated with inflated responsibility accounts for 6% of the variance of responsibility, 14% of the variance of threat estimate and 10% of the variance of perfectionism of obsessive beliefs. However, external criticism both directly and indirectly and through beliefs associated with inflated responsibility accounts for 7% of the variance of importance of obsessive beliefs.

Finally, bootstrapping using 1000 re-sampling was used to investigate the mediating role of beliefs associated with inflated responsibility, size and significance of indirect effects of external criticism of parents on obsessive beliefs. The coefficient and significance of these effects are presented in Table 4.

**Table 4:** Indirect effects of external criticism of parents on obsessive beliefs through these beliefs

Indirect effect	Coefficient	bootstrapping		sig

		Upper limits	Lower limits	
**External criticism on Responsibility via RR**	0.33	0.06	0.01	0.004
**External criticism on Threat estimation via RR**	0.03	0.06	0.01	0.003
**External criticism on Threat estimation via OP**	0.01	0.04	0.004	0.02
**External criticism on Threat estimation via AI/C**	0.03	0.06	0.01	0.001
**External criticism on Perfectionism via RR**	0.04	0.08	0.02	0.001
**External criticism on Perfectionism via OP**	0.01	0.03	0.002	0.04
**External criticism on Importance via RR**	0.03	0.06	0.01	0.003
**External criticism on Importance via AI/C**	0.02	0.04	0.001	0.04

Significance of indirect effects is assessed through bootstrapping method with respect to upper and lower limits of the effects, which represents the range of indirect effect in 1000 re-samplings. If the upper and lower limits do not include zero, indirect effect is significant (Preacher & Hayes, 2008). Thus, according to the results of Table 4, all indirect effects of the model are significant at the level of 0.05. These results show that beliefs associated with inflated responsibility can mediate the relationship between external criticism and obsessive beliefs.

## 4. Discussion

The study examined the hypothesis of the mediating effect of responsibility beliefs on the relationship between external criticism of parents and obsessive beliefs of adolescents. The results showed that there is a significant relationship between external criticism and all aspects of responsibility construct and importance subscale of obsessive beliefs (Tables 1 and 2). In obsessive-compulsive cognitive model, the experience of systematic criticism or blame has a significant role in inflamed sense of responsibility so that it is assumed that the criticism substantially expressed by parents increases the acceptance of responsibility. It should be noted that this does not mean that criticism alone is sufficient to create a growing sense of responsibility (P. M. Salkovskis et al., 1999). In susceptible individuals, casual increase in the responsibility may lead to a growing sense of responsibility. In such situations, the individual may take additional commitments or duties that the commitments could put him or her forward to greater responsibility. The likelihood of developing OCD may rise after accidental or critical events in people who have had a profound sense of responsibility since childhood (or in children and adolescents who have had strongly protective but critical parents) (Rhe-Âaume, Freeston, LeÂger, & Ladouceur, 1998) However, the recognition of the disorder varies according to the different origins of responsibility.

The results of this study were consistent with other research that have shown responsibility beliefs (by some mechanisms) with OC disorder symptoms in different nonclinical samples of children and adolescents (Ghassemzadeh et al., 2005; Reynolds & Reeves, 2008; Williams, Salkovskis, Forrester, & Allsopp, 2002) and in line with other findings (Baptista, Magna, & Mckay, 2011; Julien, O’Connor, Aardema, & Todorov, 2006; Wolters et al., 2011), indicating the importance of attention to thoughts and beliefs in areas of obsessive-compulsive disorder. Cognitive model of obsessive-compulsive disorder identifies types of primary experiences that may predispose an individual to development of the disorder. Salkovskis (P. M. Salkovskis, 1996) in his theory states that criticism by parents as a violation to and influence on children through the influence of structures of inflated responsibility, can increase the symptoms of obsessive-compulsive disorder in them. This model emphasizes the role of criticism and demanding parenting practices as a factor causing the spread of obsessive beliefs and behaviors. For example, obsessive behavior may be created as techniques to obtain parents satisfaction and avoid being criticized (Cameron, 1947) also extensive research has shown that increasing levels of criticism in the family have been associated with obsessive-compulsive symptoms in adults and children (Leonard et al., 1993; Tynes, Salins, & Winstead, 1990). parents who criticized highly cause higher mental costs to mistakes for adolescents and this leads them to work harder to do things properly and avoid mistakes and this leads to controlling and obsessive behaviors (Davies, 2005), perfectionism style may also be appeared as an attempt to gain confirmation rather than criticism from others so that such strategies can lead to obsessive-compulsive behaviors (such as controlling behaviors) to avoid criticism. Criticism based on the characteristics of responsibility can lead to an inflamed sense of responsibility and therefore potentially increase obsessive-compulsive behaviors (Rachman, 2002).

The assumed relationship between responsibility, criticism and obsessive-compulsive behaviors is evaluated indirectly in some studies through assessment of negative feedback effects (Mancini & D’Olimipio, 2004). According to Rachman’s theory (Rachman, 2002) if those who show obsessive behaviors stress on a precautionary purpose, a true experience of negative feedback increases their motivation and thus inspecting behaviors increase and automatic thoughts may appear on damage and then inspecting and obsessive behaviors rise again. The relationship between this issue and mediation of thoughts related to responsibility has been proven in several studies (Lopatka & Rachman, 1995; Sonia, Thwaites, & Freeston, 2011; Van-Dijk & Kluger, 2004).

## 5. Conclusion

With regard to the discussed issues it can be concluded that demanding parenting styles by applying authority and no criticism could avoid raising a child who has obsessive cognitions. In this regard, previous experiences of criticism may be useful in efforts to understand levels of responsibility and perfectionism that influence the formation of obsessive thoughts. The preliminary and cautious study showed criticism can be involved in the formation of obsessive thoughts but in order to identify potential affecting mechanisms of criticism on obsessive-compulsive disorder, further experimental research is needed. As well as to examine other aspects and mechanisms associated with OC disorder, a comparison between individuals with the disorder and controls with anxiety and without anxiety and with major depression seems useful and helpful.

The limitations of this study include the following: Limiting the age range from 15 to 18 years due to the variety of age ranges of children, difficulty of getting support from schools and teachers during the research as well as the large number of questions that could be (with respect to the emotions of the age of the students) effective on responses.
